# Forest stand productivity derived from site conditions: an assessment of old Douglas-fir stands (*Pseudotsuga menziesii* (Mirb.) Franco var. *menziesii*) in Central Europe

**DOI:** 10.1007/s13595-019-0805-3

**Published:** 2019-02-20

**Authors:** Tamara Eckhart, Elisabeth Pötzelsberger, Roland Koeck, Dominik Thom, Georg J. Lair, Marcela van Loo, Hubert Hasenauer

**Affiliations:** 1 Institute of Silviculture, University of Natural Resources and Life Sciences, 1190 Vienna, Austria; 2 alpS GmbH, 6020 Innsbruck, Austria; 3 Rubenstein School of Environment and Natural Resources, Burlington, VT 05405 USA; 4 Institute of Soil Research, University of Natural Resources and Life Sciences, 1190 Vienna, Austria; 5 Department of Botany and Biodiversity Research, University of Vienna, 1030 Vienna, Austria

**Keywords:** Non-native tree species, Climate change adaptation, Site conditions, Site index

## Abstract

*****Key message***:**

**Douglas-fir growth correlates with the climate, the soil moisture regime, and the soil nutrient status, reflecting a broad physiological amplitude. Even though planting this non-native tree species is suggested as a viable strategy to improve adaptiveness of European forests to a more extreme climate and to assure future productivity, the expected temperature increase may induce a decline in forest stand productivity for Douglas-fir in already warm and dry regions.**

**Context:**

Tree species selection is one of the most important forest management decisions to enhance forest productivity and stand stability on a given site. Douglas-fir (*Pseudotsuga menziesii* (Mirb.) Franco var. *menziesii*), a non-native species from north-western America, is seen as an important additional species option for adapting Central European forests to a changing climate.

**Aims:**

This study assesses Douglas-fir forest productivity derived from site conditions. We investigate climatic and physico-chemical soil characteristics and productivity of 28 mature Douglas-fir stands growing on siliceous, as well as carbonate bedrock material in southern Germany and north-eastern Austria.

**Methods:**

The importance of climatic and physico-chemical soil characteristics was analyzed with the machine learning method *Random Forests*.

**Results:**

The results show that Douglas-fir growth correlates with climate, soil moisture, and soil nutrient availability derived from ten climatic and physico-chemical soil parameters.

**Conclusion:**

The broad pH optimum between 4.5 and 7.2 reflects the broad physiological amplitude of Douglas-fir, and no significant differences were detectable between carbonate and siliceous bedrock. We also conclude that climate change may induce a forest stand productivity decline, because lower productivity with the highest mean summer temperature across our study range was observed at the warmest sites in Eastern Austria.

**Electronic supplementary material:**

The online version of this article (10.1007/s13595-019-0805-3) contains supplementary material, which is available to authorized users.

## Introduction

Douglas-fir (*Pseudotsuga menziesii* (Mirb.) Franco) is a native tree species in western North America, which was introduced to Europe almost 200 years ago (Bastien et al. 2013). Currently, Douglas-fir covers more than 800,000 ha of which 50% are in France, 25% in Germany, and the remaining 25% are distributed across other European countries. In Germany and Austria, 2% and 0.2%, respectively, of the total forest area within the country are covered with Douglas-fir (Englisch 2008; Kownatzki et al. 2011). In its native range, Douglas-fir covers an area from British Columbia (Canada) to Mexico (2200 × 4500 km). The species has adapted to different ecological conditions, resulting in different growth patterns and varying phenological traits (Gould et al. 2012; Lavender and Hermann 2014). Two distinct varieties of Douglas-fir are known: (i) the coastal variety (*P. menziesii* var. *menziesii*) and (ii) the interior variety (*P. menziesii* var. *glauca*) (Eckenwalder 2009). Its excellent growth performance across a wide range of site conditions, the high wood quality, and its resistance towards diseases and insects have made the species one of the most important commercial tree species in the world (Bastien et al. 2013). Based on the growth performance, provenances recommended for Central Europe come from the Cascades in Washington and Oregon (Kleinschmit and Bastien 1992; Weißenbacher 2008; Bastien et al. 2013). The interior variety is generally not recommended for Central Europe due to lower growth rates and a higher susceptibility to Swiss needle cast (Boyle 1999; Bastien et al. 2013) and therefore is not very common in this region (Hintsteiner et al. 2018). *Pseudotsuga menziesii* var. *menziesii* is however widely regarded as an especially promising option to increase productivity and to adapt European forests to climate change (Spiecker et al. 2019). With the increasing promotion of Douglas-fir, also concerns about potential negative ecological impacts are expressed from nature conservation side. Studies of ecological consequences of Douglas-fir cultivation in Europe were reviewed by Schmid et al. (2014). This review concludes that in Europe, Douglas-fir regenerates naturally especially on poor sites (dry, acidic), where it is not outcompeted by native tree species, but the ecological impacts of Douglas-fir seem to be minor compared to other non-native trees (e.g., *Robinia pseudoacacia* L. in Europe). Nevertheless, Douglas-fir can cause changes in species composition.

Within its natural distribution range, Douglas-fir grows on a wide range of soils and different parent materials, including marine sandstones and shales in the coastal region of northern California, Oregon, and Washington as well as soils of glacial origin in southwestern British Columbia. The soils of the northern range of the Cascades are derived from metamorphosed sedimentary material, while igneous rocks and formations of volcanic origin are important in the southern Cascades. The highest growth rates are reported on well-aerated soils with a pH value ranging from 5 to 6. Poorly drained or compacted soils inhibit Douglas-fir growth (Lavender and Hermann 2014). Climatic variation in the natural range is large. The coastal variety in the Pacific Northwest experiences a maritime climate with mean annual precipitation rates of 760–3000 mm, and a more continental climate towards the east of the Cascades (annual precipitation 600–3000 mm) (Lavender and Hermann 2014). Compared to the climate in Central Europe, most of the precipitation occurs in winter (Englisch 2008), and the summer period is relatively dry.

In Central Europe, Douglas-fir has mainly been introduced on well-drained, aerated, and carbonate-free soils. Since the beginning of Douglas-fir management in Europe, a lot of attention has been given to the carbonate content, as Douglas-fir growing on calcareous soils often shows leaf yellowing (chlorosis) (Englisch 2008). Furthermore, on sites with high manganese concentration, symptoms of toxicity have been reported (LWF 2008). However, little information is available on the growth performance across different soil types in Europe. This lack of knowledge is problematic, as for an active forest transformation, where tree species sensitive to climate change are replaced by better adapted native or non-native trees (Bolte et al. 2010), Douglas-fir is seen as one of the most promising options and is therefore more and more planted by forest owners. Consequently, with the increasing interest in planting Douglas-fir, there is also higher interest of forest owners in better understanding the site-suitability of Douglas-fir to avoid costly cultivation failures. In the case of this non-native tree species, which has been first introduced to Europe in 1827, a limited number of mature stands still exist. These stands can be analyzed in retrospect to determine the site impact on the productivity potential. In this paper, we investigate a wide range of physico-chemical soil and climatic characteristics to assess the productivity of 28 mature Douglas-fir stands growing on soils developed on siliceous and carbonate bedrock in southern Germany and north-eastern Austria. We are interested in deriving the forest stand productivity across different site conditions by assessing the importance of specific climatic and physico-chemical soil characteristics for the recorded growth performance of even-aged mature Douglas-fir stands. As a proxy of forest stand productivity, the site index at the age of 60 years was used. Because of the strong interdependency of impacts of climate and soil on forest growth, the aim was to derive the effect sizes and independent effects of climate and soil parameters on the site index with an advanced statistical method, the *Random Forests* regression approach. The objectives of this study were (i) to determine the dominant climatic and physico-chemical soil parameters and (ii) to show their effect size and correlation with the forest stand productivity of Douglas-fir in Central Europe.

## Material and methods

### Study sites

**Fig. 1:**
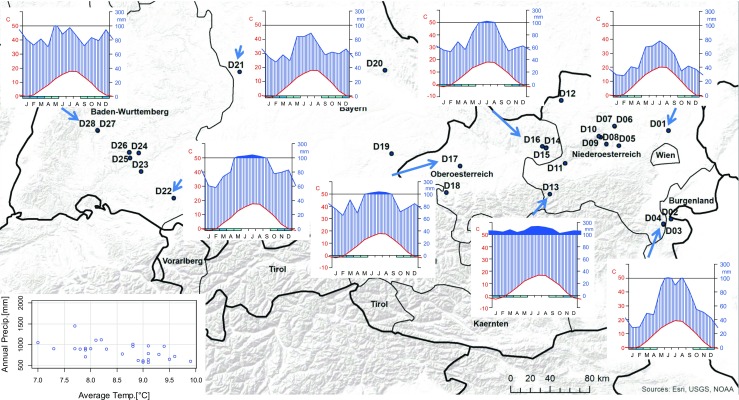
Location of all 28 Douglas-fir sites, climatic region (mean annual temperature and mean annual precipitation), and selected climate diagrams which are representative for the different regions for the years 1981–2010

In 28 even-aged Douglas-fir stands with (i) stand age 40–120 years, (ii) stand size ≥ 1 ha, and (iii) proportion of Douglas-fir basal area ≥ 80%, tree data, physico-chemical soil data, and climatic data were collected. The forest stands are located over a 600-km-long and 150-km-wide band north-east of the Alps, stretching across three Austrian provinces (Burgenland, Lower Austria, Upper Austria) and two German provinces (Baden-Württemberg, Bavaria) (see Fig. 1). With these forest stands, we cover a wide range of site, climatic, geological, and topographic variation in Central Europe (Table 1).

**Table 1:** Investigated Douglas-fir stands (D01–D28): site index (SI, height at the age of 60 years), annual precipitation (Annual Precip.), mean annual temperature (Annual Temp.), elevation in meters above sea level and geology

Site	SI	Annual Precip.	Annual Temp.	Elevation	Geology
	(m)	(mm)	(°C)	[m]	
D01	28.2	600	9.9	290	Loess
D02	36	720	9.6	460	Boulders in a sand-loam matrix
D03	36.9	790	9.1	560	Mica schist, quartz phyllonite
D04	31.1	770	9.3	520	Muscovite gneiss
D05	34.4	650	9.5	360	Sand and argillaceous marl
D06	29.6	570	9.1	370	Granulite
D07	25.3	580	9	400	Granulite
D08	34	640	9.1	430	Granulite
D09	39	620	8.9	440	Migmatized granite-gneiss
D10	36.6	610	9	410	Paragneiss, locally mica schist (partly migmatized)
D11	36	960	9.4	330	Rubble
D12	30.3	710	7.9	530	Granite
D13	41.7	2100	7.1	820	Carbonate-free, fine sandstone, arkose, slate, stone coal
D14	39.7	900	8	640	Granite
D15	41.6	910	7.9	660	Granite
D16	39.9	890	8.3	590	Granite
D17	34.8	1111	8.1	660	Gravel in sand matrix, fluvial
D18	34.6	1450	7.7	810	Sandstone, calcareous marl
D19	38.9	960	8.8	480	Silt, clayey-sandy, often gravelly
D20	34	900	7.3	680	Biotite-granite
D21	37.5	780	8.6	480	Impact breccia
D22	38.5	1120	8.2	670	Glacial till, silt, sand, gravel and stones
D23	42.1	870	7.9	660	Gravel, silt, clay, often stones, rubble
D24	30.7	900	7.7	700	Limestone, dolomite
D25	31.1	890	7.8	700	Sponge-stromatolite-corallian limestone
D26	36.7	1050	7	890	Limestone, dolomite
D27	37.3	970	9.1	450	Limestone, dolomite
D28	35.4	1010	8.8	520	Dolomite

In the drier areas of Eastern Austria (Burgenland, parts of Lower Austria), annual precipitation ranges from 570 to 790 mm, and the mean annual temperature ranges between 8.9 and 9.9 °C. In Southern Germany and along the northern border of the Austrian Alps, the conditions are more humid with an annual precipitation between 810 and 2100 mm and a mean annual temperature between 7.0 and 9.1 °C.

All selected forest stands originate from the coastal and western Cascade region of Oregon and Washington. The native origin of the Douglas-fir stands was analyzed in a recent study by Hintsteiner et al. (2018) and/or was confirmed by the forest owners. Hintsteiner et al. (2018) assigned 26 out of 28 study sites to only one genetic cluster, cluster I, and two sites to the directly adjacent genetic cluster II (Fig. S1). These genetic clusters (cluster I and cluster II) comprise the most important Douglas-fir provenances, which are recommended for the study area in Germany and Austria and for which a similar growth performance in provenance trials has been determined (Kleinschmit et al. 1979; Ruetz 1981; Schultze and Raschka 2002; Chakraborty et al. 2016). Hence, we were able to minimize any potential genetic differences in the growth performances, and the bias genetic differences may have induced in assessing the forest stand productivity.

### Forest data

In each of the 28 Douglas-fir stands, individual tree data were collected based on four angle-count sampling plots with a basal area factor of 4 (see Bitterlich 1948). For each tree, we recorded the diameter at breast height (dbh), height, age, position, and tree species. The dominant tree height by species of the angle-count sample plots was calculated as the mean height of the three trees with the largest dbh (Pollanschütz 1971). Stand age was determined by coring and counting the year-rings of the mean diameter tree of the angle-count sample.

The site index (SI) for all 28 Douglas-fir stands was defined as the mean dominant tree height at age 60 years (Kindermann and Hasenauer 2005). This site index at 60 years (SI60) was iteratively calculated according to the dominant height growth function after Mitscherlich/Richard (1919) for “Douglas-fir north-western Germany DoNwd” (Kindermann and Hasenauer 2005):

1 $$ \mathrm{SI}=a{\left(1-{e}^{-b\ast t}\right)}^c $$

2 $$ a={a}_0+{a}_1\ast {SI}_{60}+{a}_2\ast SI{2}_{60} $$

3 $$ b={b}_0+{b}_1\ast {SI}_{60}+{b}_2\ast SI{2}_{60} $$

4 $$ c={c}_0+{c}_1\ast {SI}_{60}+{c}_2\ast SI{2}_{60} $$

where, SI is the mean dominant tree height, SI60 is the dominant tree height at the age of 60 years, *t* the stand age, and *a*, *b*, and *c* the coefficients for “Douglas-fir north-western Germany (DoNwd)” based on the yield table from Bergel (1985) (see Table S1).

### Climate data

Site-specific climate information for the years 1981 to 2010 was derived with the climate interpolation tool DAYMET, which had been validated and adapted for Austria, using the national climate station network (several hundreds of stations) from Austria and Germany (Thornton et al. 2000; Petritsch 2002; Hasenauer et al. 2003). With DAYMET, daily minimum and maximum temperature (Tmin, Tmax) and precipitation (Prcp) were interpolated to each site from surrounding, 25 (temperature) and 15 (precipitation) climate stations, respectively, and daily mean temperature (Tmean) was calculated as the mean of Tmin and Tmax (Table 1). For more details on the interpolation routine, see Petritsch and Hasenauer (2014).

### Soil sampling and properties

Humus layers and mineral soil horizons were determined according to the “Guidelines for Forest Site Mapping in Austria” (Englisch and Kilian 1999) for each of our 28 Douglas-fir stands. Mineral soil samples were extracted with a Pürckhauer type gauge auger. Diagnostic soil horizons were separated and described according to depth, horizon boundaries, texture, rock content, color, concretions, carbonate presence, structure, rooting intensity, and earthworm activity.

At each plot, 11 sub-samples in 1-m distance along a diagonal transect were collected to a depth of 35 cm with a gauge auger. The 11 soil samples were separated into layer A (A horizon) and B (mineral soil), mixed and homogenized in a bucket before further use. At each stand, four separate soil profiles, each derived from 11 sub-samples, were analyzed. The bulk density for layers A and B was measured in undisturbed soil samples taken with metal cylinders (volume 100 cm^3^). The composite (mixed) samples were air-dried and sieved to 2 mm. We used the weight of the macroscopic organic material (including roots) and rocks to calculate the coarse fragment content in the air-dried soil sample.

Soil acidity (pH value) was determined in a H_2_O saturation extract with a soil-to-solution ratio of 1:2.5 as described in the Austrian standard ÖNORM L 1083 (1989). Total carbon of the bulk soils was measured after dry combustion as described in ÖNORM L 1080 (1999). The carbonate content of the soil samples was measured by the Scheibler method (see ÖNORM L 1084, 1989) to calculate the content of inorganic C. Organic carbon was then calculated as the difference of total and carbonate carbon. The determination of total nitrogen (N) was carried out with the method after Kjieldahl as described in the Austrian standard ÖNORM L 1082 (2009), and the carbon to nitrogen ratio (C/N) is the organic carbon content divided by the total nitrogen content. The exchangeable cations (Ca, Mg, K, Na, Al, Fe, Mn, H) were measured according to the Austrian standard ÖNORM L 1086 (2001), extracting 5 g of soil with 100 ml 0.1 M BaCl_2_ solution. Effective cation-exchange capacity (CEC eff.) and base saturation (BS) were calculated as the sum of the exchangeable cations and the percentage of the CEC occupied by the basic cations K, Na, Ca, and Mg. The anion nutrients nitrate (NO_3_^−^), nitrite (NO_2_^−^), phosphate (PO_4_^3−^), and sulfate (SO_4_^2−^) were determined in a H_2_O saturation extract as described in ÖNORM L 1092 (2005), extracting 5 g of soil with 50 ml H_2_O solution. The water extraction determines the anion nutrients in the soil solution, thus dissolved of readily soluble forms. The soil particle size distribution was determined by use of wet-sieving and sedimentation with the pipette analysis (Köhn pipette) after adding H_2_O_2_ to remove organic matter and dispersing with 50 ml sodium pyrophosphate (ÖNORM L 1061, 2002). For each soil parameter, we calculated the arithmetic mean of the four samples per layer, collected in one stand. The cation and anion nutrients, total nitrogen, and carbon were expressed as soil stocks on a mass per unit area basis using bulk density, coarse fragment content, and depth of layers A and B (0–35 cm). Pore volume (PV) was calculated using bulk density and a particle density following Osman (2013). The water holding capacity at field capacity, defined as − 0.015 MPa, was calculated based on soil depth and sand, silt, and clay percentages using empirical pedo-transfer functions of Clapp and Hornberger (1978) and Cosby et al. (1984) (Table S1, Eqs. 1–5).

### Geology data

The investigated Douglas-fir stands grow on soils developed on siliceous (D01–D23) and carbonate bedrock (D24–D28) (see Table 1). The exact geology for the Austrian stands was extracted from geological maps of the Geological Survey of Austria (GBA), if available 1:50,000 otherwise 1:200,000 maps for Upper Austria (Oberösterreich) and Lower Austria (Niederösterreich). The geology for the German stands was extracted from 1:200,000 geological maps of the German Federal Institute for Geosciences and Natural Resources (BGR).

### Statistical analysis of Douglas-fir productivity versus site parameters

The site index defined as the mean dominant tree height at age 60 is used as a measure for forest stand productivity. Thus, we correlate the site index (Table 2) to a set of 25 climatic and physico-chemical soil parameters determined for each of the 28 Douglas-fir stands (D01–D28–see Table 2). We used the statistical method *Random Forests* (Breiman 2001) to select the number of predictors and derive their effect size on the site index. *Random Forests* fits decision trees according to hierarchical levels. Subsequently, all individual trees (weak learners) are aggregated to a random forest (strong learner) using the bootstrap approach, where about 63% of the original observations are used for the prediction and the remaining 37% are “out-of-bag” observations that determine the accuracy and error rates of the predictions (e.g., cross-validation). In contrast to many other statistical methods (e.g., AIC and BIC), *Random Forests* measures the importance of a variable directly using the misclassification rate of the out-of-bag observations (Cutler et al. 2007).

**Table 2:** Summary of the dependent variable and the 25 candidate variables of the 28 Douglas-fir stands for statistical analysis with *Random Forests*. Climate variables refer to the summer months June, July and August. Soil variables are the depth-weighted mean values of layers A and B. Geology relate to calcareous site or silicate site

Variable group	Acronym	Description	Unit	Mean	Min	Max
Site index	SI	Mean dominant tree height at age 60 years	m	35.4	25.3	42.1
Climate	Tmean	Mean summer temperature [JJA]	°C	17.4	15.8	19.3
	Psum	Summer precipitation [JJA]	mm	313	219	690
Soil	pH H_2_O	Actual pH	[−]	5.0	4.0	7.8
	C	Carbon	t/ha	43	14	119
	N	Nitrogen	t/ha	3	0.5	9
	C/N	C/N ratio	[−]	18	11	30
	Ca	Calcium	kg/ha	3929	23	25,298
	Mg	Magnesium	kg/ha	312	3	2508
	K	Potassium	kg/ha	108	22	250
	Fe	Iron	kg/ha	18	0	76
	Al	Aluminum	kg/ha	592	0	1859
	Mn	Manganese	kg/ha	72	0.1	275
	CEC eff.	Cation-exchange capacity	mmol/kg	157	40	657
	Bsat	Base saturation	%	47	10	100
	NO_3_^−^	Nitrate	kg/ha	13	0.4	58
	NO_2_^−^	Nitrite	kg/ha	1	0	6
	PO_4_^3−^	Phosphate	kg/ha	1	0	10
	SO_4_^2−^	Sulfate	kg/ha	13	1	69
	Clay	Clay	%	19	6	47
	Sand	Sand	%	36	0	60
	Skeleton	Soil skeleton	%	14	1	40
	PV	Pore volume	%	69	50	84
	Soil depth	Effective soil depth	cm	82	33	149
	WHC	Water holding capacity	mm	275	128	565
Discrete variable group	Description		Unit	Allocation		
Geology	Carbonate or siliceous bedrock		Dummy (1/0)	5 sites (1), 23 sites (0)		

*Random Forests* was used since it (i) is a non-parametric method, which is able to illustrate saturation levels as well as optimum ranges of key site factors driving Douglas-fir growth, (ii) has a high prediction accuracy even if predictor variables are moderately collinear (Dormann et al. 2013), and (iii) can deal with different variable types. The statistical analysis was carried out in two main steps.

I. Variable pre-selection: First, we eliminated irrelevant predictors using the *Random Forests*–based variable selection procedure of the VSURF package in R (Genuer et al. 2015) and optimized the number of variables randomly sampled as candidates for each tree using the tune RF function of the *Random Forests* package (Liaw and Wiener 2015). This function uses different numbers of candidate variables to fit the regression trees. The model with the lowest out-of-bag error indicates the best estimate.

II. Building the final *Random Forests* model: We fitted the *Random Forests* model using 2000 regression trees. We used the mean square error (MSE) as a measure of variable importance, which indicates the decrease in model accuracy by randomly permuting the observations of a variable. Partial dependence plots illustrate the marginal effect of each explanatory variable on site index variation while averaging other variable effects (Cutler et al. 2007).

## Results

**Fig. 2:**
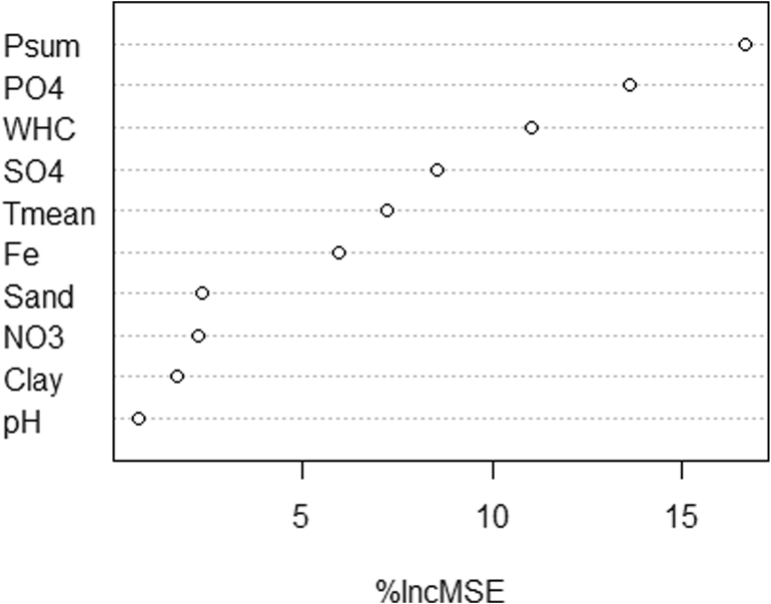
Variable importance plot (all other variables were not meaningful), where the variable importance is expressed as the percentage increase in mean square error (%IncMSE). The mean square error (MSE) indicates the loss of predictive power of the same model by omitting a variable. Variable elimination using VSURF (step 1: preliminary elimination and ranking). The variables summer precipitation (Psum, mm), phosphate (PO4, kg/ha), water holding capacity (WHC, mm), sulfate (SO4, kg/ha), mean summer temperature (Tmean, °C), iron (Fe, kg/ha), sand content (%), nitrate (NO3, kg/ha), clay content (%), and pH value explain 30.3% of the variance (pseudo *R*^2^ = 0.303)

**Fig. 3:**
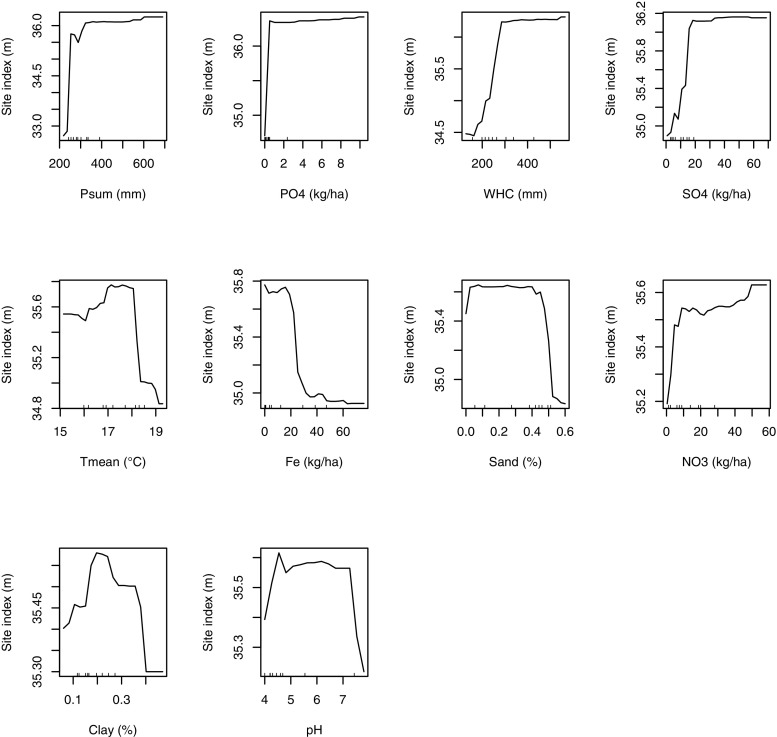
Partial effect plots based on results from the *Random Forests* analysis, showing the mean marginal influence of ten explanatory variables summer precipitation (Psum), phosphate (PO4), water holding capacity (WHC), sulfate (SO4), mean summer temperature (Tmean), iron (Fe), sand content, nitrate (NO3), clay content, and pH value on site index variation. Each plot represents the effect of the explanatory variable while holding the other variables constant

Site index as the dominant height at age 60 years (Table 1) ranged between 25.3 m (D07) and 42.1 m (D23). For the analysis with *Random Forests*, we used 25 potential explanatory variables (Table 2) from 28 observations to explain the variation in site index. From the 25 candidate variables, forward selection procedure resulted in ten variables which were further analyzed with *Random Forests*. These ten variables contained soil and climatic parameters and explained 30.3% of the variance (pseudo *R*^2^ = 0.303). Variable importance rankings revealed that summer precipitation exhibited the largest impact in explaining site index variation (Fig. 2). Next, phosphate (PO_4_^3−^) and water holding capacity (WHC) were found to be important, followed by sulfate (SO_4_^2−^), summer mean temperature, iron (Fe^3+^), sand content, nitrate (NO_3_^−^), clay content, and pH value. Partial effect plots indicated a non-linear relationship between site index and the ten explanatory variables (see Fig. 3).

Summer precipitation and water holding capacity were positively correlated with the site index. Both impacted distinctively Douglas-fir growth if values dropped below 270 mm and 300 mm, respectively. Higher summer precipitation and water holding capacity values did not lead to a further improvement in site index and thus in productivity of Douglas-fir stands. The partial dependence plots also indicated that nutrient requirements of phosphate (PO_4_^3−^), sulfate (SO_4_^2−^), and nitrate (NO_3_^−^) begin to saturate at stock rates of 0.2 kg/ha, 20 kg/ha, and 10 kg/ha, respectively. Iron (Fe^3+^) stocks above 18 kg/ha showed a negative sigmoidal relationship with the site index. The impact of mean summer temperature on the site index was optimal between 17 and 18 °C and decreased above 18 °C.

**Fig. 4:**
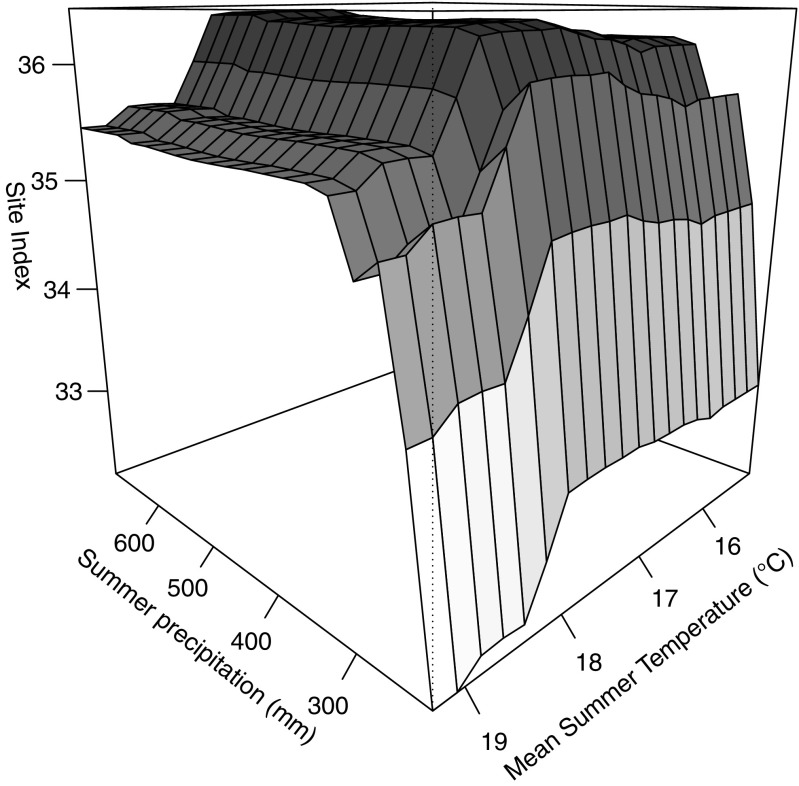
3D plot for the climate variables summer precipitation and mean summer temperature. For the prediction of the site index, all other predictors were kept constant at their mean values

Site index dropped if the sand content exceeded 45%. The clay content showed an optimal range between 18% and 26%, whereas the site index decreased with clay contents greater than 38%. The pH value showed a broad optimum between 4.5 and 7.2. The 3D plot (Fig. 4) illustrates the joint impact of climate variables, summer precipitation, and mean summer temperature. The temperature optimum is independent from the amount of precipitation (Fig. 4). High summer precipitation does not avoid a drop in the site index if mean summer temperatures exceed 18 °C. A decline in site index by 3–4 classes is evident, if the summer precipitation drops below 300 mm.

## Discussion

Studying the relationship between forest stand productivity and site characteristics has a long tradition in forest science (Aertsen et al. 2010). Many of the early studies used basic statistical methods like linear regression for the analysis (e.g., Carter and Klinka 1990, Curt et al. 2001, Fontes et al. 2010), which do not account for potential non-linearities in ecological relations and show problems with collinearity (Aertsen et al. 2010). The *Random Forests* approach, compared to traditional statistical methods can be classified as less transparent (“black box” model). It is able to identify structures in complex, often non-linear data sets (Olden et al. 2008) and is resilient to collinearity (Dormann et al. 2013). In our analysis, *Random Forests* identified ten of our 25 investigated climatic and physico-chemical soil parameters that drive Douglas-fir productivity (see Fig. 2) in Central Europe, and assessed a non-linear relationship between the predictors and the response (Fig. 3). The summer precipitation (Psum) and water holding capacity (WHC) (Fig. 2) are two of the three most important site characteristics and directly refer to the water budget. The third of the three important variables is phosphate (PO_4_^3−^), a common cause for forest productivity limitation, which is often neglected in site-growth studies (Bontemps and Bouriaud 2014).

Our results suggest that a low summer precipitation (< 270 mm) and a low water holding capacity (< 300 mm) reduce productivity (see Fig. 3). This confirms the findings by Carter and Klinka (1990) as well as Curt et al. (2001), who showed a significant correlation between Douglas-fir site index versus soil water-deficit and available soil water storage, respectively. The optimal soil water conditions for Douglas-fir can be expressed by the available water storage capacity (AWSC). The AWSC describes the portion of soil water which is accessible to plant roots, i.e., water in medium-sized pores. It can be estimated from the water holding capacity (WHC) and the soil type (Blume et al. 2010). On silty soils, a proportion of 15% for medium pores (Blume et al. 2010) can be assumed. According to the detected optimal WHC of > 300 mm, the estimated AWSC is about 45 mm and can be classified as “low” for Douglas-fir (Leitgeb et al. 2012).

Douglas-fir productivity declines if the mean summer temperature (Tmean) exceeds 18 °C (Fig. 3). This temperature sensitivity at high temperatures might be related to an increase in vapor pressure deficit, which was shown by Restaino et al. (2016) to be strongly correlated with decreased Douglas-fir growth across forests in the Western United States. The detected moderate increase in productivity with mean summer temperatures between 15 °C and 17 °C (Fig. 3) supports the findings of Jansen et al. (2013), who found higher growth rates at lower elevations. These findings are important in the context of expected climate change, since global mean annual temperatures are projected to increase between 1.1 °C (RCP 4.5) and 4.8 °C (RCP 8.5) by 2100 (IPCC 2014). Accordingly, water stress may become an important limitation for productivity due to a combined effect of changes in summer temperature and summer precipitation (Fig. 4) on the warmest sites in Eastern Austria (D01–D11, mean summer temperature > 18 °C) (Fig. 1, Table S3). Higher soil phosphate (PO_4_^3−^), sulfate (SO_4_^2−^), and nitrogen (NO_3_^−^) contents show an increase in Douglas-fir productivity (Fig. 3). Phosphorus and nitrogen are often the main limiting nutrients for plant growth (Lambers et al. 1998). Crop production studies show a link between sulfur and nitrogen, which are often co-limiting (Hawkesford and De Kok 2006; Fageria 2014). If the iron (Fe^3+^) concentration exceeds 18 kg/ha, site productivity dropped (see Fig. 3). This confirms that iron plays a crucial role in metabolic processes, but may turn toxic if critical accumulation levels are exceeded (Rout and Sahoo 2015).

The physical soil properties sand and clay content lead to varying soil fertility and productivity. While on sandy soils (sand content > 45%) and clayey soils (clay content > 38%), a decline in Douglas-fir growth is evident, a moderate clay content improved productivity (see Fig. 3). Sandy soils drain more water and are poor in fertility as well as water supply (Osman 2013). Soils with clay content above 40%, which are classified as clayey soils (Blume et al. 2010), retain large amounts of water but drain very slowly. Thus they are often waterlogged (Osman 2013), which induces damages of the fine root system of Douglas-fir (Englisch 2008; Lavender and Hermann 2014). Rout and Sahoo (2015) found that under waterlogged and acidic soil conditions, iron may be taken up excessively leading to damages of vital cellular constituents in plants.

Our study included five Douglas-fir stands growing on carbonate soils (see Table 1). Although current management recommendations in Austria and Germany suggest planting Douglas-fir on carbonate-free soils (e.g., Englisch 2008), no significant decline in productivity (e.g., site index) between our old Douglas-fir stands on carbonate soils (SI: μ = 34.2 m) versus siliceous soils (SI: μ = 35.7 m) was detectable (see Table 3). This finding was also supported by our analysis with *Random Forests*, where the dummy variable “carbonate” or “siliceous bedrock” (1/0) did not enter the final model (Fig. 2, Table 2). Within this context, it has to be highlighted that the forest soils of all five Douglas-fir stands on carbonate sites show high loam contents, and the results cannot be extrapolated, e.g., to Rendzina sites. The carbonate sites showed phosphorus below the detection limit (Table 3), which is typical for soils with pH values above 7 as phosphorus is fixed as calcium phosphate (Rowell 1994). Phosphorus is a rather immobile element in the soil compared to other nutrients and its availability is pH dependent. However, plants are able to change the pH value in the rhizosphere by root exudation to mobilize P of calcium phosphates (George et al. 2012). Moreover, mycorrhizal associations to enhance plant P uptake (Lambers et al. 2006) may supply the trees especially in cases of very low readily available phosphate content. Considering these mechanisms for increasing P-availability and the potentially different relative importance of the other site parameters on carbonate sites, the measured zero value of PO_4_^3−^ on carbonate sites does not contradict our finding of the overall high importance of phosphate. Essentially, with the selection of our studied forest stands, we did not intend to explore the very limits of Douglas-fir growth for any specific site parameter. Our study design of investigating old Douglas-fir stands, however, revealed ecologically meaningful non-linear relations over a wide range of site qualities where Douglas-fir has been planted during the last century.

**Table 3:** Mean value and standard deviation (sd) of site index (SI) and the ten influencing site variables grouped by geology: Calc. group refer to calcareous sites (*n* 5) and silic. group to silicate sites (*n* 23)

	Calc. group		Silic. group	
Variables	Mean	sd	Mean	sd
Site index (SI)	34.2	3.1	35.7	4.5
Summer precipitation (Psum)	287.8	26.1	319.7	103.8
Phosphate (PO_4_^3−^)	0.0	0.0	1.3	2.8
Water holding capacity (WHC)	255.2	92.2	280.7	114.2
Sulfate (SO_4_^2−^)	2.9	1.3	14.9	14.7
Mean summer temperature (Tmean)	16.3	0.9	17.5	1.0
Iron (Fe)	0.0	0.0	20.9	21.9
Sand content	4%	3%	41%	12%
Nitrate (NO_3_^−^)	17.6	6.5	12.8	14.5
Clay content	31%	9%	17%	5%
pH value	7.5	0.2	4.4	0.4

## Conclusion

Douglas-fir growth correlates with the climate, the soil moisture regime, and the soil nutrient status. The observed pH optimum between 4.5 and 7.2 corresponds to the broad physiological amplitude of Douglas-fir. Even though the non-native tree species Douglas-fir is more drought resistant than our main native species, the expected temperature increase (e.g., higher summer temperatures) will also induce a decline in forest stand productivity for Douglas-fir. A lower productivity due to mean summer temperatures above 18 °C was observed at the warmest sites in Eastern Austria. Important macronutrients for Douglas-fir growth are phosphate, sulfate, and nitrogen; whereas high contents of the micronutrient iron reduce growth. The negative impact of clayey soils (clay content > 38%) on Douglas-fir growth could be confirmed in our study, as they are often waterlogged. Despite the current recommendations in avoiding Douglas-fir plantation on calcareous soils, our study showed no significant differences in growth performance on carbonate and siliceous bedrock. However, it is important to note that our study covered only five out of 28 forest sites on rather loamy carbonate soils. We suggest that further Douglas-fir sites on carbonate soils need to be investigated.

## Electronic supplementary material

ESM 1 (DOCX 590 kb)

## Data Availability

The dataset generated during and/or analyzed during the current study is available in the Figshare repository Eckhart et al. (2019) DF_site_growth_data. V1. FigShare. [Dataset]. 10.6084/m9.figshare.7553384.v1
