# Supplementary material for: Forest stand productivity derived from site conditions: an assessment of old Douglas-fir stands (Pseudotsuga menziesii (Mirb.) Franco var. menziesii) in Central Europe
Source: Ann For Sci. 2019 Feb 20;76(1):19. doi: 10.1007/s13595-019-0805-3 (PMC6394740; doi:10.1007/s13595-019-0805-3)
Supplement: Supplementary file 1 — (DOCX 590 kb) [file 13595_2019_805_MOESM1_ESM.docx]

**Supplementary material**


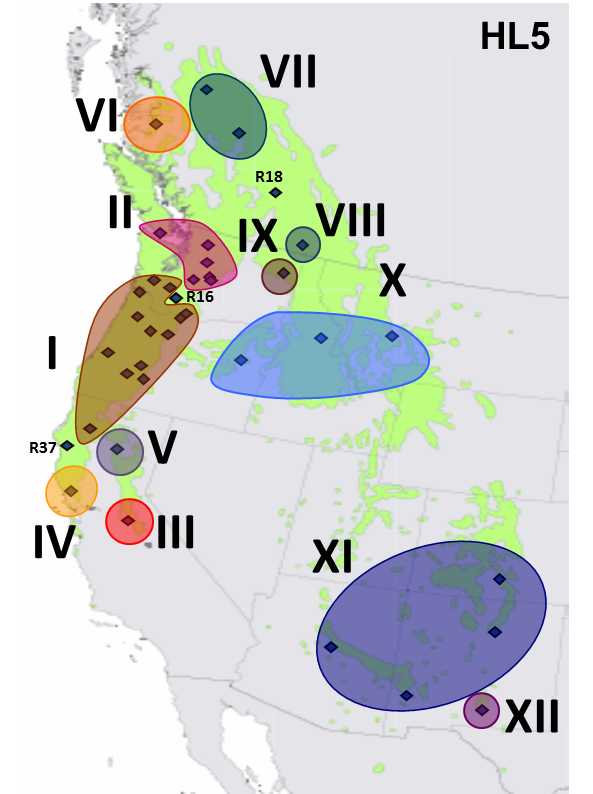


**Cluster I: D01, D03-D08,**

**D10-D28**

**Cluster II: D02, D09**

**Fig. S1** Distribution map of the hierarchical cluster level 3 (HL3) for 38 Douglas-fir populations within its natural range in Northwest America. HL3 contains 12 differentiated genetic clusters (I-XII). Cluster I-VI refer to the coastal variety, Cluster VII-XII to the interior variety. Three reference populations (R16, R18, R37) are cluster-admixed populations. Study populations (D01-D28) were assigned to the coastal and western Cascade region in Oregon and Washington (Cluster I-II) (Hintsteiner et al., 2018).

**Table S1** Coefficients for the dominant tree height function after Mitscherlich/Richard (1919) for “Douglas-fir northwestern Germany (DoNwd)” (Kindermann and Hasenauer, 2005).

|  | **a0** | **a1** | **a2** | **b0** | **b1** | **b2** | **c0** | **c1** | **c2** |
| --- | --- | --- | --- | --- | --- | --- | --- | --- | --- |
| **DoNwd** | -5.92E+00 | 1.26E+00 | 0 | 9.90E-02 | -2.79E-03 | 2.54E-05 | 9.43E+00 | -3.15E-01 | 3.03E-03 |

**Table S2** Equations for the soil water content at field capacity.

| **Equations** | | **Parameters** | |
| --- | --- | --- | --- |
| 1 | $\Psi_{sat}=-\mathrm{e}^{\left[\left(1.54-0.0095\cdot P_{sand}+0.0063\cdot P_{silt}\right)\cdot\log(10)\right]}\cdot9.8\text{e}-5$ | *Ψ_sat_*  (MPa) | Soil water potential at field capacity |
| 2 | $\theta_{sat}=\frac{(50.5-0.142\cdot P_{sand}-0.037\cdot P_{clay})}{100}$ | *Θ_sat_, Θ_fc_* | Volumetric water content at saturation and at field capacity |
| 3 | $b=-3.10-0.157\cdot P_{clay}+0.003\cdot P_{sand}$ | *b* | Empirical shape parameter |
| 4 | $\theta_{fc}=\theta_{sat}\left(\frac{-0.015}{\Psi_{sat}}\right)^{\frac{1}{b}}$ | *P_sand_, P_silt_, P_clay_* (%) | Percentage of sand, silt and clay (Σ=100) |
| 5 | $W_{fc}=1000\cdot d_{soil}\cdot\theta_{fc}$ | W_fc_  (mm) | Soil water content at field capacity |
|  |  | *d_soil_*  (m) | Soil depth |

**Table S3** SI (Site index) and the 10 influencing site variables Psum (Summer precipitation), PO4 (Phosphate), WHC (Water holding capacity), SO4 (Sulfate), Tmean (Mean summer temperature), Fe (Iron), Sand, NO3 (Nitrate), Clay, pH value.

| **Site** | **SI** | **Psum** | **PO4** | **WHC** | **SO4** | **Tmean** | **Fe** | **Sand** | **NO3** | **Clay** | **pH** |
| --- | --- | --- | --- | --- | --- | --- | --- | --- | --- | --- | --- |
|  | **(m)** | **(mm)** | **(kg/ha)** | **(mm)** | **(kg/ha)** | **(°C)** | **(kg/ha)** | **(%)** | **(kg/ha)** | **(%)** | **(-)** |
| **D01** | 28.2 | 219 | 0.3 | 259 | 6 | 19.3 | 32 | 43 | 9.1 | 12 | 4.3 |
| **D02** | 36 | 274 | 0.1 | 229 | 13 | 18.7 | 11 | 44 | 6.1 | 13 | 4.5 |
| **D03** | 36.9 | 307 | 0.5 | 212 | 10 | 18.1 | 1 | 42 | 8.0 | 14 | 4.4 |
| **D04** | 31.1 | 301 | 0.5 | 194 | 2 | 18.4 | 44 | 60 | 0.4 | 6 | 4.2 |
| **D05** | 34.4 | 259 | 0.0 | 337 | 6 | 18.7 | 1 | 12 | 17.9 | 28 | 4.7 |
| **D06** | 29.6 | 237 | 0.4 | 206 | 10 | 18.4 | 52 | 42 | 0.9 | 12 | 4.2 |
| **D07** | 25.3 | 239 | 1.0 | 158 | 8 | 18.2 | 4 | 52 | 6.2 | 16 | 4.9 |
| **D08** | 34 | 258 | 0.0 | 154 | 4 | 18.2 | 40 | 51 | 1.3 | 10 | 4.2 |
| **D09** | 39 | 252 | 0.4 | 245 | 5 | 18.0 | 5 | 41 | 8.8 | 11 | 4.6 |
| **D10** | 36.6 | 249 | 0.1 | 226 | 5 | 18.1 | 6 | 36 | 1.7 | 12 | 4.6 |
| **D11** | 36 | 338 | 0.0 | 260 | 4 | 18.5 | 2 | 27 | 1.8 | 16 | 4.1 |
| **D12** | 30.3 | 281 | 0.1 | 244 | 14 | 16.8 | 37 | 50 | 3.1 | 15 | 4 |
| **D13** | 41.7 | 690 | 0.4 | 337 | 11 | 15.8 | 1 | 11 | 15.6 | 24 | 5.9 |
| **D14** | 39.7 | 330 | 0.4 | 300 | 18 | 16.9 | 17 | 46 | 25.7 | 16 | 4.4 |
| **D15** | 41.6 | 332 | 0.6 | 279 | 15 | 16.8 | 17 | 49 | 7.8 | 19 | 4.3 |
| **D16** | 39.9 | 325 | 0.4 | 305 | 18 | 17.2 | 47 | 45 | 18.7 | 22 | 4 |
| **D17** | 34.8 | 388 | 0.3 | 128 | 22 | 16.9 | 48 | 55 | 33.9 | 16 | 4 |
| **D18** | 34.6 | 510 | 0.0 | 518 | 12 | 16.1 | 76 | 33 | 13.5 | 22 | 4.3 |
| **D19** | 38.9 | 345 | 0.1 | 565 | 15 | 17.6 | 4 | 26 | 5.7 | 22 | 5 |
| **D20** | 34 | 293 | 0.0 | 413 | 16 | 16.0 | 31 | 48 | 1.3 | 18 | 4 |
| **D21** | 37.5 | 245 | 5.5 | 188 | 69 | 17.2 | 5 | 50 | 40.1 | 23 | 4.2 |
| **D22** | 38.5 | 396 | 8.5 | 238 | 14 | 16.7 | 1 | 39 | 9.2 | 17 | 4.6 |
| **D23** | 42.1 | 285 | 10.4 | 462 | 46 | 16.3 | 0 | 46 | 58.3 | 16 | 4.5 |
| **D24** | 30.7 | 288 | 0.0 | 229 | 3 | 16.0 | 0 | 0 | 20.0 | 47 | 7.5 |
| **D25** | 31.1 | 286 | 0.0 | 145 | 3 | 16.1 | 0 | 9 | 19.5 | 25 | 7.8 |
| **D26** | 36.7 | 331 | 0.0 | 311 | 4 | 15.1 | 0 | 2 | 22.5 | 29 | 7.4 |
| **D27** | 37.3 | 265 | 0.0 | 382 | 1 | 17.3 | 0 | 6 | 6.1 | 27 | 7.4 |
| **D28** | 35.4 | 269 | 0.0 | 210 | 3 | 17.0 | 0 | 4 | 19.9 | 27 | 7.6 |
